# Identifying circumstances under which high insecticide dose increases or decreases resistance selection

**DOI:** 10.1016/j.jtbi.2017.06.007

**Published:** 2017-09-07

**Authors:** J.C. Helps, N.D. Paveley, F. van den Bosch

**Affiliations:** a Department of Computational and Systems Biology, Rothamtsed Research, Harpenden, Hertfordshire AL5 2JQ, UK; b ADAS High Mowthorpe, Duggleby, Malton, North Yorkshire YO17 8BP, UK

**Keywords:** Insecticide resistance, Modelling, Simulation model, Dosimetry, Target-site

## Abstract

Insect management strategies for agricultural crop pests must reduce selection for insecticide resistant mutants while providing effective control of the insect pest. One management strategy that has long been advocated is the application of insecticides at the maximum permitted dose. This has been found, under some circumstances, to be able to prevent the resistance allele frequency from increasing. However this approach may, under different circumstances, lead to rapid selection for resistance to the insecticide.

To test when a high dose would be an effective resistance management strategy, we present a flexible deterministic model of a population of an insect pest of agricultural crops. The model includes several possible life-history traits including sexual or asexual reproduction, diploid or haplodiploid genetics, univoltine or multivoltine life cycle, so that the high dose strategy can be tested for many different insect pests. Using this model we aim to identify the key characteristics of pests that make either a high dose or a low dose of insecticide optimal for resistance management. Two outputs are explored: firstly whether the frequency of the resistance allele increases over time or remains low indefinitely; and secondly whether lowering the dose of insecticide applied reduces or increases the rate of selection for the resistance allele.

It is demonstrated that with high immigration resistance can be suppressed. This suppression however, is rarely lost if the insecticide dose is reduced, and is absent altogether when individuals move from the treated population back into an untreated population. Reducing the dose of insecticide often resulted in slower development of resistance, except where the population combined a high influx of less resistant individuals into the treated population, a recessive resistance gene and a high efficacy, in which case reducing the dose of insecticide could result in faster selection for resistance.

## Highlights

• A model is presented that simulates the control of agricultural insect pests.
• The model tracks the selection of resistance under different doses of insecticide.
• In most plausible scenarios reducing the dose of insecticide reduces selection.

## 1 Introduction

Insecticides place a selection pressure on insect pests of agricultural crops to evolve resistance, and resistance has developed in many pest species against the major insecticidal modes of action currently on the market (Tabashnik et al., 2014). Management strategies (and in particular insecticide application strategies) that can slow down or prevent the build-up of resistance in agricultural insect pests are therefore desirable.

Ideally the choice of a resistance management method should be based on experimental evidence. Due to the difficulties in experimental assessment of the effect of an insecticide resistance management method (field studies are often too noisy to discern differences e.g. (Castle et al., 2002, Parker et al., 2006), while laboratory studies typically use a small population size that cannot test the same strategies that would be used in a field), recourse is often taken to the development and analysis of mathematical models. Models are able to simulate a wider range of management options than individual field or laboratory experiments, and over longer time scales. The use of different strength doses of insecticides (Roush and Tabashnik, 1990, Tabashnik and Croft, 1982), mixtures of insecticides (Comins, 1986, Mani, 1985), and alternations of insecticides (Mani, 1989) have all been explored using computational models as potential strategies that could reduce the rate at which resistance develops in an insect pest population.

One resistance management topic that is debated is that of the dose of insecticide that should be applied, in particular whether the application of a high dose will lower or raise selection for resistance. Current guidance (IRAC, 2013) is that foliar applied insecticides should be applied at the dose rate recommended on the product label (the maximum permitted dose) to reduce the rate of build-up of resistance. A general and convincing reason for this was put forward by Georghiou and Taylor (1977). They reasoned as follows: consider the situation where resistance against the insecticide is developing but its frequency is still small. In such situations almost all resistance alleles will reside in heterozygote individuals. This is simply because virtually all individuals carrying one or more resistance alleles (be it SR or RR) will mate with a homozygote sensitive individual (SS). The result of such mating is heterozygous individuals (and homozygote sensitive individuals). Since the vast majority of the resistance alleles in the population are in heterozygotes, and heterozygotes are partially sensitive to the control chemical, it makes sense to apply a high dose as this will kill heterozygote individuals and thus remove R alleles from the population thereby delaying (or even preventing) an increase in the fraction of individuals carrying one or more resistance alleles. The above reasoning resulted in the implementation of the high-dose refuge strategy for delaying the resistance to Bt crops (Roush, 1997) which is thought to have had some success (Tabashnik et al., 2013).

The above reasoning applies where the insect pest species is both diploid and sexually reproducing. The reasoning does not apply to clonal species, such as *Myzus persicae*, an aphid which is parthenogenetic in the UK. In clonal species the few homozygote resistant individuals will give rise to further homozygote resistant offspring and have a considerable selective advantage in the presence of the mode of action, so cannot be stopped from invading by the high dose strategy. Surprisingly this has not been recognised in the published evidence on insecticide resistance management.

A further reason why the high dose strategy may not be appropriate in relation to foliar insecticide applications, is the assumption that a high enough dose of insecticide can be applied to ensure the death of heterozygote individuals, or to reduce the size of the population to a level where chance might lead to the extinction of resistant strains. While such levels of control might be a reasonable assumption for transgenic plants expressing insecticides, they are unlikely to be achievable even with the maximum permitted dose of foliar insecticides. Because insecticides can have adverse impacts on non-target organisms, regulators have to ensure that the maximum permitted dose on the product label is also the minimum dose required to achieve effective control (Anon, 2012). The process of determining that dose was described by Finney (1993), as follows. In farm crops, there are many factors that affect the level of control achieved with any given dose. Hence, if dose-response experiments are repeated across many sites and seasons, many different dose-response curves result. For practical purposes, the label recommended dose is usually set at the level which gives a high level of control in a high proportion of circumstances. Finney suggested 80–90% control in 80–90% of field experiments as a typical aim, although higher levels of control may be required for insect pests, for example, to prevent insect contamination in horticultural crops. There are two consequences of this process of determining the full label dose which are relevant to the analysis presented here. Firstly, the label dose will not be set at a level which gives 100% control, even of sensitive strains, since, as previously mentioned, this is not the aim of the maximum allowed dose specified on a product label. Hence, the modelling approach presented assumes that it is not possible to drive resistant strains to extinction by insecticide treatment, within the range of doses permitted. Secondly, there will be circumstances (for example, when pest pressure is moderate) when commercially acceptable levels of control may be obtained with less than the full label dose. Fungicide doses below the label recommended dose are routinely used on crops in many countries (Jørgensen et al., Press) and a similar approach may be economically advantageous for insecticide use, provided the use of lower doses does not adversely affect resistance management.

In this paper we explore, with the use of a mechanistic model, under what conditions of a pest's life cycle and genetics lowering the dose of a foliar-applied insecticide can lead to an increase or a decrease in the build-up of resistance. We explore this for a monogenic target-site resistance gene, which is the primary resistance mechanism underlying many cases of resistance failure, for example the failure of pyrethroids (Bass et al., 2014, Foster et al., 2014). The methods may not apply to resistance caused by metabolic resistance processes, nor when managing two or more target-site resistance genes simultaneously. We simulate a range of life cycles including diploid and haplodiploid insect species as well as clonally reproducing and sexually reproducing insect species.

The authors realise that resistance management is only one aspect that needs to be considered when developing an insecticide application program to control an insect crop pest, and may be in many cases not the determining consideration – the efficient control of the pest in question will be in most cases the main consideration. Therefore, although our aim is to understand the effect of dose on the selection for resistance, the results must be scrutinised for the key requirement that the dose applied must be appropriate to provide effective control under the particular circumstances of pest pressure in the field.

The aim of the paper is to identify which life-cycle characteristics of agricultural insect pests and which pesticide attributes result in either a full dose (as specified on the insecticide label) or a reduced dose being the optimal management strategy, in order to delay the development of insecticide resistance for as long as possible. In doing this we attempt to clarify for which sorts of agricultural foliar insect pests and insecticides it is beneficial to use the highest dose possible each application, and for which pest-insecticide combination it is better to reduce the dose as much as is feasible.

## 2 Methods

We describe below the insect model used to test the effect of management strategies on the resistance of an agricultural insect pest population. The population, consisting of up to four stages of insect development (egg, larva, pupa and adult), is modelled in continuous time over several seasons, with an overwintering phase between each season (details below).

In order to illustrate which insect life history characteristics or pest-pesticide interactions determine whether high or low doses are best in order to limit the development of resistance, we carry out parameter searches on a model parameterisation for a generic insect population which is not parameterised for any specific insect, but has parameters that are comparable to a range of aphid pests (see Table 1 for parameters). This population is hemimetabolous—only larvae and adults are modelled—and local parameter searches are carried out from this typical insect model. However, to demonstrate the results carry over for specific insects the model was also parameterised for three particular agricultural crop insects of economic importance with different life histories: *Myzus persicae*, the peach-potato aphid; *Meligethes aeneus*, the pollen beetle; and *Frankliniella occidentalis*, western flower thrips. These represent a variety of population dynamics; peach-potato aphid is an asexual (in the UK) multivoltine insect with only larvae and adults and a pest of many outdoor and protected crops, pollen beetle is a sexual univoltine insect that is an important pest of oilseed rape, and western flower thrips is a multivoltine sexual haplodiploid insect found mainly in protected environments. All three species have developed resistance, and the intercept and gradient of the dose response were found for each species for a particular insecticide resistance (see Appendix A and Table 1).

**Table 1:** Descriptions and values for the model variables and parameters. Details for each parameter value can be found in Appendix 1.

Variable / parameter	Description	Default value	*M. persicae*	*M. aeneus*	*F. occidentalis*	Unit
***I***_0_	Initial density of insects each season	0.5	0.5	0.5	0.1	(insect plant^-1^)
***K***	Threshold density of the population	20	50	30	10,000,000	(insect plant^-1^)
***β***	Insect birth rate at low densities	0.1333	0.75	4	0.55	(insect day^-1^)
***η***	Rate of emergence from overwinter population	0.0	0.1	0.25	0.0	(day^-1^)
***ξ***	Insecticide decay rate	0.5	0.5	0.5	0.5	(day^-1^)
***RR***_0_	Initial proportion of homozygote resistant individuals	1e-8	1e-8	1e-8	1e-8	(1)
***SR***_0_	Initial proportion of heterozygote individuals	1e-4	1e-4	1e-4	1e-4	(1)
***I***_***I***_	Immigration: untreated to treated population	0.0	0.01	0.01	0.0	(day^−1^)
***ι***_***E***_	Emigration: treated to untreated population	0.0	0.01	0.01	0.0	(day^−1^)
***κ***	Relative size of the untreated population	2.0	2.0	2.0	2.0	
***μ***_***E***_	Egg lifespan	NA	NA	5	6.7	(day)
***μ***_***L***_	Larvae lifespan	10	7	7	9.8	(day)
***μ***_***P***_	Pupa lifespan	NA	NA	12	5.2	(day)
***μ***_***A***_	Adult lifespan	20	10	12	18.0	(day)
***ω***_***E***_	Natural mortality of eggs	NA	NA	0.01	0.0	(insect day^-1^)
***ω***_***L***_	Natural mortality of larvae	0.0	0.2	0.025	0.0	(insect day^-1^)
***ω***_***P***_	Natural mortality of pupae	NA	NA	0.01	0.0	(insect day^-1^)
***ω***_***A***_	Natural mortality of adults	0.0	0.2	0.01	0.0	(insect day^-1^)
tSpray	Day on which insecticide is sprayed	50	50	5	50	(day)
***a*** [SS,SR,RR]	Intercept of logit-dose lines for each genotype	[0.5, −2, −4]	[2.5,0.85,−0.5]	[12.0,7.95,3.9]	[−1.0,−2.5,−6.0]	
***b*** [SS,SR,RR]	Gradient of logit-dose lines for each genotype	[1.5, 1.5, 1.5]	[2.0,2.0,2.0]	[1.5,1.5,1.5]	[2.5,2.5,2.5]	
***ϕ***	Dominance of the resistance gene	0.25	0.45	0.5	0.5	(1)
	The number of days in a single season	200	100	60	70	(days)

In the following sections we first describe the simulation of the pest population within a crop growth season, before explaining the details of the implementation of sexual or asexual reproduction of a diploid or haplodiploid population, the assumptions made concerning the overwintering population, and the effect of insecticides on the population.

### 2.1 Model outline

The model keeps track, through consecutive seasons, of the densities of agricultural insect pests (per crop plant or per unit area, for example) that are susceptible or resistant to an insecticide. Each season insects emerge from an overwintering population, and then develop for one or more generations during a season. The model can describe both diploid and haplodiploid insects (for a haplodiploid insect population we divide the population into males and females: females are diploid, males are haploid). Resistance to the insecticide is determined by a single locus. This is a common case in insecticide resistance usually leading to virtually absolute resistance of the homozygote resistant individuals. Other types of resistance such as metabolic or detoxifying resistance are not our model target. In diploid insects we therefore model three possible resistance genotypes: homozygote susceptible (SS), heterozygote (SR), and homozygote resistant (RR) for the insecticide resistance gene, in both larvae (L) and adults (A) of the insect. For haplodiploid insects we additionally model the male haploid genotypes being either susceptible (S) or resistant (R) in both larvae and adults. The mean duration of each stage in the absence of other sources of mortality is denoted by *μ_Stage_* days respectively, where 1/*μ_Stage_* is therefore the rate of transition from that stage to the next. Natural mortality from external sources occurs at rate *ω_Stage_* for each stage. Adult insects give birth to eggs or larvae (depending on whether the insect has a full life cycle (holometabolous) or only a partial life cycle (hemimetabolous)) at rate $\beta {\left(1-\frac{E+L+P+A}{K}\right)}^{+}A$, such that the birth rate is zero when the total density of insects reaches a threshold, *K*; this is constrained so that the number of births cannot be negative.

The system of equations for a diploid insect with a full life cycle is summarised as:

$$\frac{dE_{GG}}{dt}=\;\beta {\left(1-\frac{\sum_{GG}T_{GG}+\;\sum_{G}T_{G}}{K}\right)}^{+}A_{TT}p_{GG}-\frac{1}{\mu_{E}}E_{GG}-\omega_{E}E_{GG}$$

$$\frac{dL_{GG}}{dt}=\frac{1}{\mu_{E}}E_{GG}-\frac{1}{\mu_{L}}L_{GG}-\omega_{L}L_{GG}-g\left(D\right)$$

$$\frac{dP_{GG}}{dt}=\frac{1}{\mu_{P}}L_{GG}-\frac{1}{\mu_{P}}P_{GG}-\omega_{P}P_{GG}$$

$$\frac{dA_{GG}}{dt}=\frac{1}{\mu_{P}}P_{GG}-\frac{1}{\mu_{A}}A_{GG}-\omega_{A}A_{GG}-g\left(D\right)+\iota_{I}\kappa U\left(t\right)\theta_{GG}-\iota_{E}A_{GG}$$

where subscript *GG* denotes the genotype under consideration (either SS, SR, or RR), and subscript *T_GG_* denotes the sum of all stages of genotype *GG. p_GG_* denotes the proportion of all offspring that result in genotype *GG* (see Section 2.1.3 Reproduction, below), and $\iota_{I}\kappa U\left(t\right)\theta_{GG}-\iota_{E}A_{GG}$ is the term for immigration (*ι_I_*) from and emigration (*ι_E_*) to a population outside the crop fields under insecticide treatment (see Section 2.1.2 below). *g*(*D*) represents the insecticide-induced mortality, and can be found in Section 2.1.4. The emergence from an overwintering population has not been included in the system of equations, but the term $+\eta O_{GG}$ is added to whichever stage overwinters for the insect being simulated (details can be found in see Section 2.1.1). To modify this to a hemimetabolous insect, the egg and pupal stages are removed, and transition from the larval stage goes to the adult stage, and the adult gives birth to larval offspring:

$$\begin{array}{ccc} \frac{dL_{GG}}{dt} & = & \beta {\left(1-\frac{\sum_{GG}T_{GG}+\;\sum_{G}T_{G}}{K}\right)}^{+}A_{TT}p_{GG}\\ & & -\;\frac{1}{\mu_{L}}L_{GG}-\omega_{L}L_{GG}-g\left(D\right) \end{array}$$

$$\frac{dA_{GG}}{dt}=\frac{1}{\mu_{L}}L_{GG}-\frac{1}{\mu_{A}}A_{GG}-\omega_{A}A_{GG}-g\left(D\right)+\iota_{I}\kappa U\left(t\right)\theta_{GG}-\iota_{E}A_{GG})$$

If the insect being simulated is haplodiploid, then the following system of equations is also simulated for each haploid genotype, *G*:

$$\frac{dE_{G}}{dt}=\;\beta {\left(1-\frac{\sum_{GG}T_{GG}+\;\sum_{G}T_{G}}{K}\right)}^{+}A_{T}p_{G}-\frac{1}{\mu_{E}}E_{G}-\omega_{E}E_{G}$$

$$\frac{dL_{G}}{dt}=\frac{1}{\mu_{E}}E_{G}-\frac{1}{\mu_{L}}L_{G}-\omega_{L}L_{G}-g\left(D\right)$$

$$\frac{dP_{G}}{\mathrm{dt}}=\frac{1}{\mu_{L}}L_{G}-\frac{1}{\mu_{P}}P_{G}-\omega_{P}P_{G}$$

$$\frac{dA_{G}}{\mathrm{dt}}=\frac{1}{\mu_{P}}P_{G}-\frac{1}{\mu_{A}}A_{G}-\omega_{A}A_{G}-g\left(D\right)+\iota_{I}\kappa U\left(t\right)\theta_{G}-\iota_{E}A_{G})$$

The number of generations per year of this insect population can be adjusted by altering the mean lifespans of each stage of the insect, with a shorter lifespan giving more generations per season. In order to model only one generation per year (a univoltine insect), the transition between the stages may be severed, either by setting β=0, so that adults do not give birth to larvae, or by setting $1/\mu_{Stage}=0$, so that one stage does not transition to the next stage. In each case the final stage will transition instead into a separate overwintering population ensuring only one generation per year.

#### 2.1.1 Overwintering

Many agricultural insect pests in temperate climates overwinter in a dormant stage either in areas of the crop that are not exposed to insecticide (primarily before an insecticide is applied) or outside the cropping area. In spring the insects emerge from this overwintering stage. To model this we simulate an overwintering compartment containing the three genotypes (*O_GG_*). At the end of each season the proportion of each genotype in the overwintering insect stage is recorded. The total density of the overwintering insect stage is the same at the start of each season. This is a realistic description for agricultural crop pests as the population size at the start of a crop growing season is for most species not related to the population size at the end of the previous growing season. The insects emerge from this overwintering population into the within-field population over time at constant rate, *η*. The rate of emergence of each genotype of the overwintering population is therefore:

$$\frac{dO_{GG}}{dt}=-\eta O_{GG}$$

Depending on whether the insect pest overwinters as eggs, larvae, pupae or adults, this same term is an influx into the appropriate life-history stage. In the simplest form of the model we assume that the rate at which the insect emerge is infinite (η=∞), meaning that insects essentially emerge instantaneously from their overwintering location.

#### 2.1.2 Immigration from and emigration to external populations

Many agricultural insect pest populations are not closed, and there is movement between populations that are exposed to insecticide and those that are not, primarily through the movement of the mobile adult stage. This is simulated in our model through the inclusion of an external population that is not explicitly modelled. Instead, at the start of a simulation the model is run for a single year without applying any insecticide, and the density of adults is stored, *U*(*t*). This adult density is then assumed to be the density of adults in the external population. Adult insects have a given per capita probability, *ι*, of moving between the two populations, either immigrating (*ι_I_*) from the external population into the treated population, or emigrating (*ι_E_*) from the treated population into the untreated population. The resistance frequency of the untreated population is therefore dependent on the movement of adults from the treated population to the untreated population, the size of the untreated population relative to the treated population, *κ*, and also on movement from the untreated population to the treated population. The change in frequency of each genotype in the untreated population is therefore:

$$\frac{d\theta_{GG}}{dt}=\frac{\iota_{E}A_{GG}-\iota_{I}\theta_{GG}U\left(t\right)}{\kappa }$$

where *U*(*t*) specified the density of adults in an untreated population.

#### 2.1.3 Reproduction

The model incorporates both asexual and sexual reproduction, and the implementations of both are described below. For sexual reproduction we additionally consider sexual reproduction of a haplodiploid insect population. In each case we aim to determine the proportion of all offspring that are of each genotype, *p_GG_*. In an asexual (i) population, the proportions of each genotype of new larvae are at the same proportion as the adults. In a sexual (ii) population, the genotypes of the new larvae are determined according to random mating between all adults and, in a haplodiploid (iii) population the genotypes are determined from random mating of haploid males and diploid females. *p_GG_* is therefore a function of the density of the genotypes of all adults in the population, and differs depending on the reproduction strategy.

(i) *Asexual population*:
  $$p_{GG}=\frac{A_{GG}}{A_{TT}}$$
  where *A_GG_* is the density of adults of genotype *GG*, and $A_{TT}=A_{SS}+A_{SR}+A_{RR}$.
(ii) *Sexual population*

For a sexual population we assume recombination as determined by Mendelian inheritance (see Appendix B). The proportion of each genotype in new offspring is given by:
  $$p_{SS}=\frac{A_{SS}\cdot A_{SS}+A_{RS}\cdot A_{SS}+0.25\cdot A_{RS}\cdot A_{RS}}{A_{TT}A_{TT}}$$
  $$p_{SR}=\frac{2\cdot A_{RR}\cdot A_{SS}+A_{RR}\cdot A_{SR}+A_{SS}\cdot A_{SR}+0.5\cdot A_{SR}\cdot A_{SR}}{A_{TT}A_{TT}}$$
  $$p_{RR}=\frac{A_{RR}\cdot A_{RR}+A_{RR}\cdot A_{SR}+0.25\cdot A_{SR}\cdot A_{SR}}{A_{TT}A_{TT}}$$

(i) *Haplodiploid sexual reproduction*

In a haplodiploid population, males result from splitting of unfertilised female eggs. In the following *A_T_* denotes the total density of male haploids, and *A_TT_* denotes the total density of the female diploids:
  $$p_{S}=\frac{A_{SS}+0.5\cdot A_{SR}}{A_{TT}}$$
  $$p_{R}=\frac{0.5\cdot A_{SR}+A_{RR}}{A_{TT}}$$
Females result from random recombination between the haploid males and diploid females:
  $$p_{SS}=\frac{A_{S}\cdot A_{SS}+0.5\cdot A_{S}\cdot A_{SR}}{A_{T}\cdot A_{TT}}$$
  $$p_{SR}=\frac{0.5\cdot A_{S}\cdot A_{SR}+A_{S}\cdot A_{RR}+A_{R}\cdot A_{SS}+0.5\cdot A_{R}\cdot A_{SR}}{A_{T}\cdot A_{TT}}$$
  $$p_{RR}=\frac{0.5\cdot A_{R}\cdot A_{SR}+A_{R}\cdot A_{RR}}{A_{T}\cdot A_{TT}}$$

#### 2.1.4 The effect of the insecticide

**Fig. 1:**
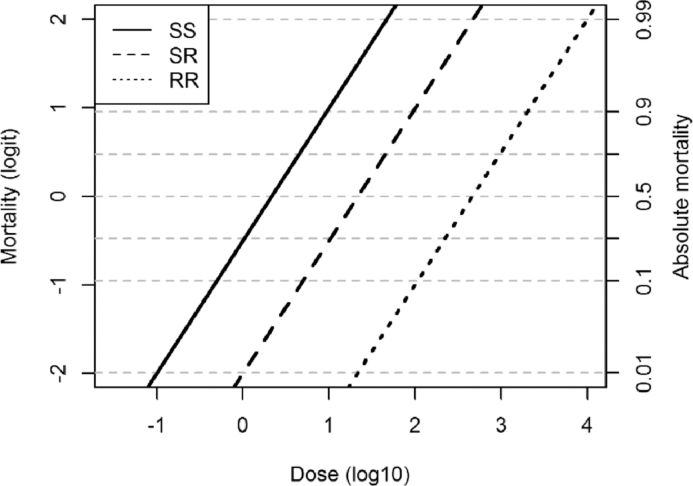
Logit mortality–log dose lines, giving the expected mortality over a single day for each insect genotype (SS, SR, RR) in a diploid insect population when exposed to a dose of insecticide over a single day, assuming the values used in the default model parameterisation (see Table 1).

We model the effect of the insecticide on each genotype as a linear relationship between the logit of the mortality for each resistance genotype within a specified time period and the log of the insecticide dose applied, which is similar in form but easier to manipulate than the traditional probit-dose curves of experimental insecticide literature. Experimental probit-dose curves (Finney, 1947) have demonstrated that the probit of insect mortality over a given time period is linearly related to the log of the applied insecticide dose. Translating the logit-dose line into a mortality rate based upon the dose of the insecticide is relatively straight-forward (see Appendix C), and results in the per capita mortality as a result of a particular insecticide dose *D* being modelled by $g\left(D\right)=\;-\log\left(1+10^{a}D^{b}\right)$, where *a* and *b* specify the intercept and gradient of one of the logit-dose lines shown in Fig. 1. In the following simulations we have assumed that the slope of the logit-dose line does not vary between genotypes, but the intercept of the logit mortality with log dose is lower for the resistant genotypes (Fig. 1). The intercept of both the susceptible and resistant homozygotes genotypes are specified (see Table 1 for parameters), and the intercept of the heterozygote is determined by the dominance of the resistance allele; $a_{SR}=\left(1-\phi \right)\cdot a_{SS}+\phi \cdot a_{RR}$, such that for a dominant resistance gene (ϕ=1) a individual with a heterozygote resistance genotype has the same logit-dose line as an individual with the homozygous resistant genotype. In order to standardise the following simulations, and to be relatable to agricultural practice, we assume that a full dose of insecticide causes a 90% reduction in the insect population (Finney, 1993), although this is varied in the parameter search. The insecticide dose decays exponentially at rate *ξ*.

$$\frac{dD}{dt}=-\xi D$$

### 2.2 Simulations

With this model we are able to simulate the changes in the frequency of each resistance genotype as a result of applying a selection pressure in the form of an insecticide, for insects with very different life cycles.

#### 2.2.1 Model output

In order to track the increase in resistant genotypes we measure the frequency of the resistance allele in the stages of the population that are susceptible to the insecticide, *f_R_*.

For a diploid population, and if we assume that all stages are susceptible to the insecticide:

$$f_{R}=\frac{0.5\left(T_{SR}\right)+\left(T_{RR}\right)}{T_{TT}}$$

where, as before, *T_SR_* denotes the density of all insect stages that are heterozygote genotype, *T_RR_* the density of all stages that are homozygote resistant, and *T_TT_* denotes the total insect population of all stages of all genotypes.

For a haplodiploid population:

$$f_{R}=\frac{0.5\left(T_{\mathrm{SR}}\right)+T_{\mathrm{RR}}+T_{R}}{T_{\mathrm{TT}}+T_{T}}$$

#### 2.2.2 Analysis

We track the frequency of a resistance allele against a specific mode of action in an agricultural foliar insect pest population to be tracked, with the aim of determining which life cycle traits and pesticide characteristics determine whether a higher or lower dose of insecticide is optimal to reduce the build-up of resistance and therefore prolong the effective life of that insecticide.

**Fig. 2:**
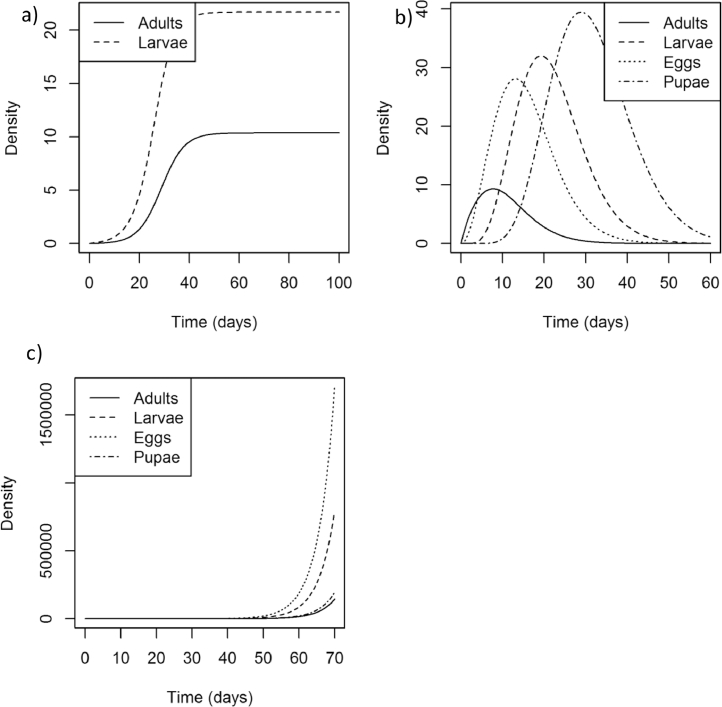
Model dynamics representing a) peach-potato aphid, b) pollen beetle, and c) western flower thrips. The parameters may be found in Table 1.

**Fig. 3:**
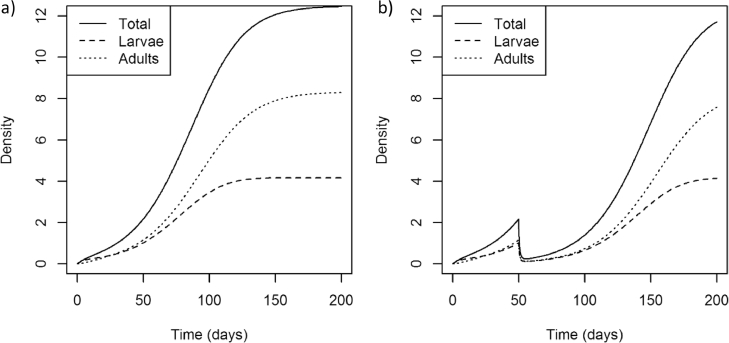
The effect of insecticide application on the insect pest model with default parameter values (see Table 1). The figures show the response of the total insect population, as well as the adults and larvae, when the population is (a) untreated, or (b) treated with a single insecticide application on day 50.

**Fig. 4:**
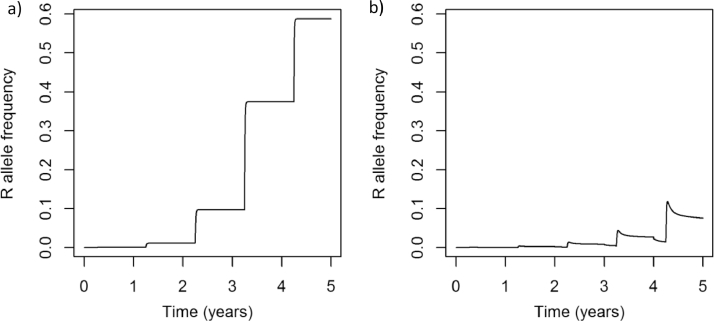
Resistance builds up over time when insecticide is sprayed each year on day 50 at the same dose with (a) the default model (see Table 1); (b) the default model parameters except that emergence from an overwintering population is included (η=0.1), as is movement between the treated and untreated populations ($\iota_{I}=0.01$, $\iota_{E}=0.01$).

Analysis of the model therefore proceeds as follows: we first show that the model is functional – both in that it can describe a variety of agricultural insect pest species (Fig. 2), and that applying a chemical control method (Fig. 3) leads under certain circumstances to the build-up of resistance to that control (Fig. 4). We next explored the model with a parameter search in order to identify parameters which could result in a high dose of insecticide giving a slower speed of selection for resistance than a reduced dose (Appendix 5). Finally, we demonstrate, for select parameters, the extent to which resistance can develop faster or slower when the dose of insecticide is reduced from a full dose to half that dose (Fig. 7).

In each simulation three factors are explored: whether applying a full dose of insecticide leads to the resistance allele increasing over time; whether decreasing the dose from a label dose leads to a change in whether the resistance allele increases from its low starting frequency; and whether reducing the dose results in an increased or decreased resistance allele frequency after 5 years (Box 1).

#### 2.2.3 Implementation

The model was written in C++, compiled with GCC 4.4.7 and run on a linux cluster. Iteration was performed by a Dormand-Prince adaptive time-step iterator (Dormand and Prince, 1980). The source code can be obtained from the corresponding author.

## 3 Results

### 3.1 Model overview

As discussed the model can describe a range of agricultural insect pest species by altering the parameters specified for a particular simulation. Fig. 2 shows simulations of each of the three insect pest species *Myzus persicae* (Fig. 2a), *Meligethes aeneus* (Fig. 2b) and *Frankliniella occidentalis* (Fig. 2c). When insecticide is applied to these populations the density of the pest population is reduced (Fig. 3) and resistance is selected for (Fig. 4).

### 3.2 Identification of critical parameters

**Fig. 5:**
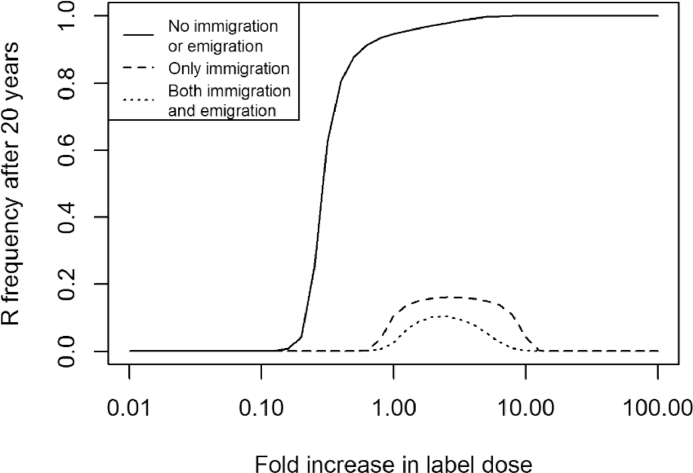
Immigration is an important factor determining whether high doses suppress the selection of resistance. Here the resistance frequency is shown after 20 years of application with a range of doses from 1% of a label dose to 100x a label dose in a diploid sexually reproducing population with default parameters (see Table 1). Three versions of movement between the untreated and treated populations are shown: no immigration or emigration ($\iota_{I}=0.0,\;\iota_{E}=0.0$), solid line; immigration from the untreated population into the treated population ($\iota_{I}=0.1$), but no emigration ($\iota_{E}=0.0$), dashed line; and both immigration ($\iota_{I}=0.1$) and emigration ($\iota_{E}=0.1$), dotted line.

In the generic parameterisation of the above insect pest model, being a hemimetabolous insect pest with no immigration or emigration, and instantaneous emergence from the overwintering phase, and with both stages susceptible to the insecticide, decreasing the dose of insecticide invariably resulted in a reduction in the rate at which resistance built up (Fig. 5), even with doses applied 100 fold higher than that giving 90% mortality. The following life history traits, however, could in certain circumstances all lead by themselves to a full dose of insecticide resulting in a lower selection rate than a reduced dose: immigration from an untreated population (Fig. 5), one or more insect stages being unaffected by the insecticide, and gradual emergence from the overwintering population (results not shown). The movement between the treated and untreated populations led to the greatest reduction in resistance at high doses.

### 3.3 Exploration of critical parameters

**Fig. 6:**
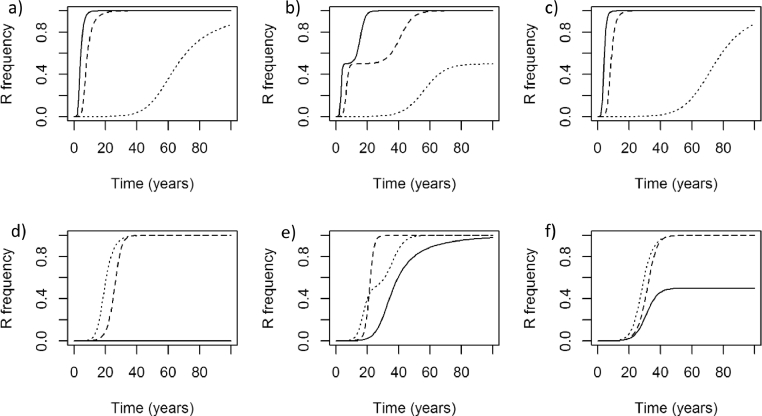
Each graph shows the resistance allele frequency over time under continuous application of three doses: full dose (solid line), 50% full dose (dashed line), 10% full dose (dotted line). In the first row the populations have the default parameter values, except with no immigration between the untreated and treated populations (Table 1). The second row has a partially recessive dominance (*ϕ* = 0.25), high immigration ($\iota_{I}=0.1$, but $\iota_{E}=0.0$) between the treated and untreated populations, and a high full dose efficacy of 97%. These combinations are shown for each reproduction strategy in each column: sexual and diploid (a) and (d); asexual and diploid (b) and (e); sexual and haplodiploid (c) and (f). Note that the parameters for the second row of simulations were selectively chosen to highlight an alternative outcome.

In Fig. 6, the typical insect model was challenged with three doses, a full dose that leads to 90% mortality after a spray, half that dose and ten percent of that dose. With no immigration entering the population the resistance frequency increased under each application dose, and inevitably increased to 100%. And, in each case, the higher dose resulted in faster selection for resistance. With the addition of immigration, however, the resistance frequency did not increase to 100% and, with a 10% dose the resistance frequency did not increase substantially above the initial resistance frequency. From the graphs in Fig. 6 it is clear that there are therefore two questions that need to be considered (Fig. 9). Firstly, when a full dose is applied does resistance increase over time or not? And secondly, when the resistance frequency increases when a full dose is applied, does lowering the dose of insecticide lead to faster or slower selection?

**Fig. 7:**
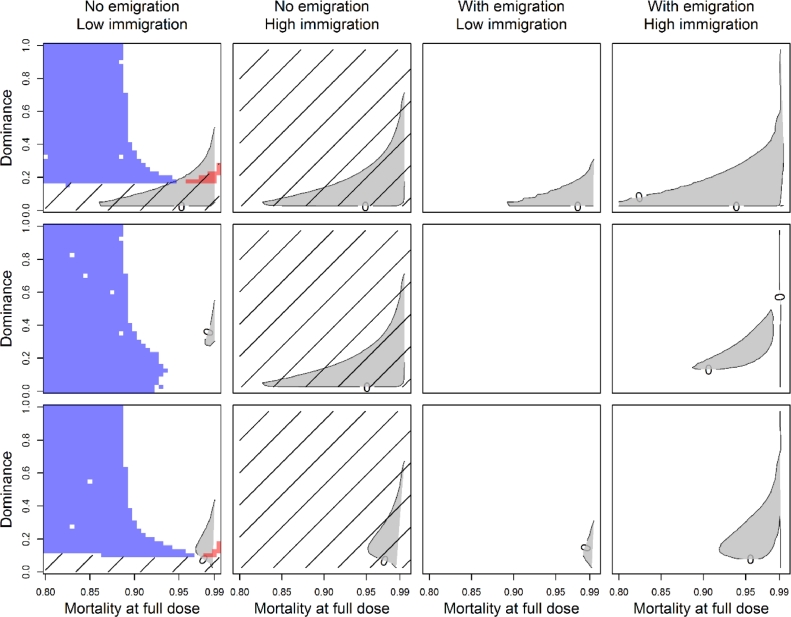
Plots exploring the range of results for each of the three reproduction strategies (each row, from top to bottom: sexual and diploid; asexual and diploid; sexual and haplodiploid) and two levels of immigration (low immigration, $\iota_{I}=0.01$; high immigration: $\iota_{I}=0.1$), and with or without emigration from the treated population (no emigration, $\iota_{E}=0.0$; with emigration, $\iota_{E}=\iota_{I}$). Each individual plot illustrates three metrics (see below and Box 1) when the mortality of a full dose of insecticide is varied from 80% to 99%, and dominance of the resistance allele is varied from fully recessive (ϕ=0.0) to fully dominant (ϕ=1.0). The three metrics depicted are: i) whether a full insecticide dose causes the R allele to not increase (area with diagonal lines); ii) whether reducing the dose from full dose to 50% of that dose leads to the R allele increasing where previously it had not (red area) or vice versa whether reducing the dose leads to the R allele becoming indefinitely low where previously it had increased (blue area); iii) whether reducing the full dose to 50% of that dose leads to an increase in the R allele frequency after 20 years (grey area). (For interpretation of the references to colour in this figure legend, the reader is referred to the web version of this article.)

The results are shown in Fig. 7 for an extended parameter search. The hashed areas represent simulations with parameters that resulted in a full dose of insecticide causing the resistance allele frequency to remain low indefinitely (below 0.05%, and not increasing) and the colours representing the possibilities depicted in the respective colours in Fig. 9.

Taking each column in turn, with low immigration from an untreated population into the treated population (1% of the population moving each day, $\iota_{I}=0.01$) and no movement from the treated population the other way (Fig. 7, left column, $\iota_{E}=0.0$), a full label dose of insecticide can suppress the development of resistance in populations with sexual reproduction, but only when the resistance allele was recessive. In the clonal population, resistance was never suppressed under low immigration. However, when the dose was reduced the lowered dose resulted in resistance not developing in the population. Only when the full dose had very high efficacy and the resistant allele was around 20% dominance did reducing the dose result in resistance increasing where it had previously not been increasing.

With high immigration (10% of the population moving each day, $\iota_{I}=0.1$) from an untreated population but still no emigration ($\iota_{E}=0.0$), applying a full dose of insecticide to the model resulted in resistance not increasing, regardless of whether the population was sexual, clonal or haplodiploid. Additionally, in all three cases reducing the dose to a half dose still resulted in resistance remaining low–suppression was not lost.

With the addition of movement of individuals from the treated population back into the untreated population at the same rate as the immigrating individuals move, resistance always developed in both populations, regardless of the dose of insecticide used.

Finally, under all three reproduction traits and with or without emigration, it was possible for the reduced dose to result in a higher resistance frequency after 20 years than the full dose. This occurred primarily when the full dose had high efficacy and the dominance of the resistance gene was mostly recessive (Fig. 7, grey area). With no emigration, reducing the dose did not result in a much more rapid increase in resistance, but with emigration, resistance could increase a lot faster with a low dose than a high dose (see Fig. 6, for example), but only in a small area of parameter space.

### 3.4 Contrasting insect pest species

**Fig. 8:**
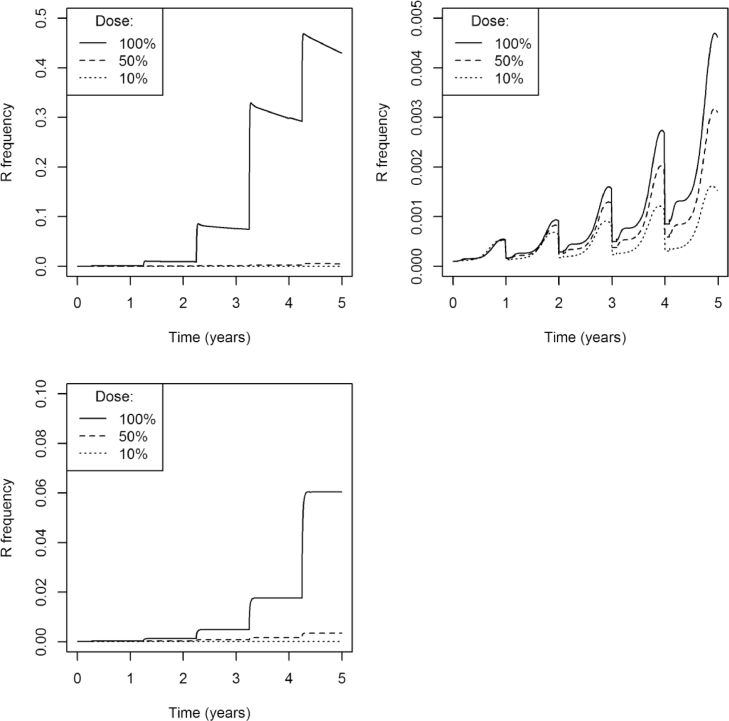
The rate of selection is shown when a single insecticide is applied every year, in three differing insect species: a) *Myzus persicae*, b) *Meligethes aeneus*, c) *Frankilinella occidentalis*. For each species three doses are compared: a full dose (100%) giving a mortality after spraying of 90% (solid line); a half dose (50%) being half of the full dose (dashed line); and a small dose (10%) being ten percent of the full dose (dotted line). Parameters can be found in Table 1.

The conclusion that reducing the dose of insecticide rarely resulted in an increase in the rate of selection for resistance was corroborated by parameterising the model for each of the three contrasting species previously mentioned: *Myzus persicae, Meligethes aeneus, Frankliniella occidentalis* (Fig. 8). Each species was controlled with either a full dose, half dose, or ten percent of the full dose. In each species, and under every dosage applied, the resistance frequency increased over time; suppression of resistance was not encountered, even at high dose. Lowering the dose invariably decreased the selection for resistance (Fig. 8).

**Fig. 9:**
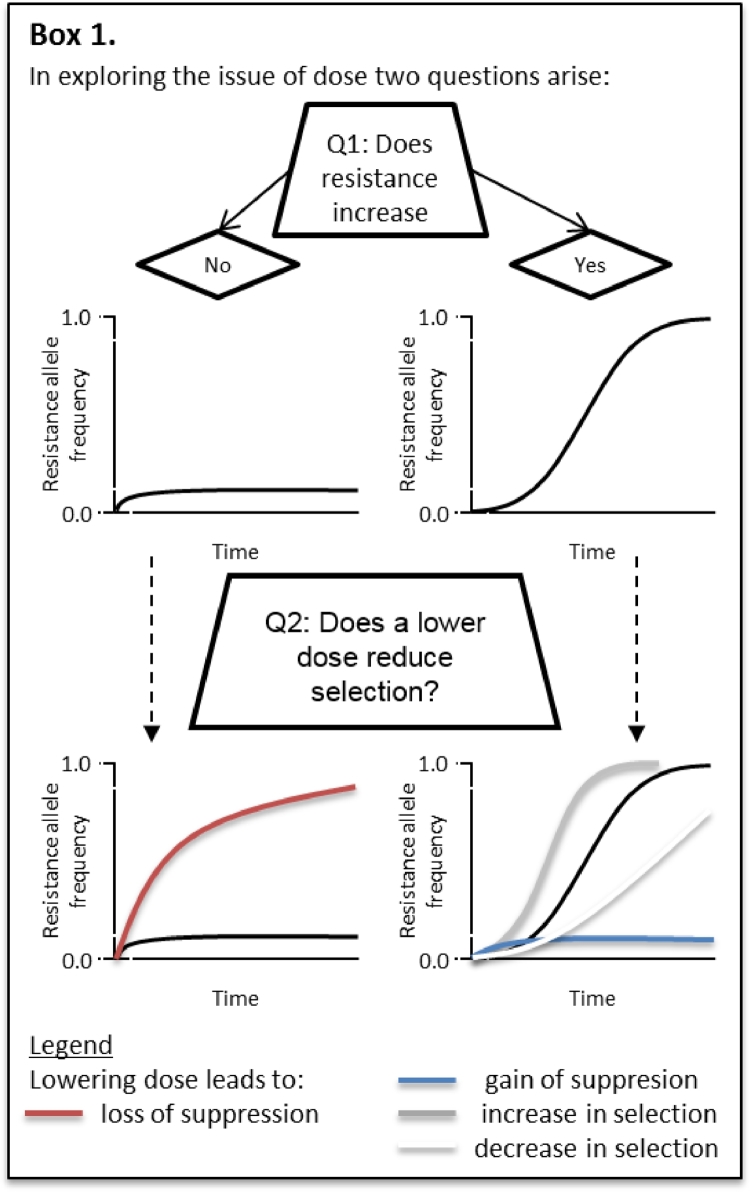
An illustration of the main questions in the paper. Firstly, dose may either increase over time or remain indefinitely suppressed when a full (label) dose of insecticide is applied annually. Secondly, in each of these cases lowering the dose could compromise or enhance resistance management. When each of these conditions may occur is depicted in Fig. 7, and the colours of the lines in Fig. 8 correspond to the colours in Fig. 7.

## 4 Discussion

The application of a high dose of insecticide has long been advocated as a strategy by which a high degree of control of an agricultural insect plant pest can be achieved whilst minimising the development of resistance. In the ideal scenario the pest can be well controlled indefinitely. Much of the modelling work has been limited in scope and complexity, largely due to computational limitations in the 70 s and 80 s when much of the work was carried out (Comins, 1977, Mani and Wood, 1984). Nevertheless Tabashnik and Croft (1982) warned that the situations in which a high dose strategy may be effective would be rare, being useful only when the insect in question had a high immigration rate, the resistance gene was functionally recessive, resistance frequency was low and the insect had low reproductive potential.

This paper considers whether lowering the dose (if this is possible without compromising effective control) would be good or bad as a resistance management strategy, and which life cycle traits or pesticide characteristics determine each outcome. The results here agree with those previously reported in many respects. In particular as others have found (Georghiou and Taylor, 1977, Rosenheim and Tabashnik, 1990, Tabashnik and Croft, 1982), although it is possible for resistance to be suppressed indefinitely it is unlikely for a biologically realistic insect population. For all three insect species modelled a full dose of insecticide resulted in resistance increasing in the population, most rapidly at the largest dose.

Our model has highlighted a key aspect of this system. For resistance to not increase over time in the model presented here, there needs to be an influx of untreated individuals into the treated population, and no emigration back into that untreated population. Without these requirements resistance always increased over time. Suppression is therefore achieved due to the dilution of the treated population by untreated individuals, so that the reversion to a susceptible population is greater than the selection imposed by the insecticide. This reasoning also demonstrates why a high dose of insecticide can slow the development of resistance more than a low dose. A high dose, by killing more of the treated population, increases the impact of any dilution from less resistant individuals, resulting in the susceptible individuals entering the population making up a greater proportion of the treated population, and therefore dilution is stronger. A valid concern therefore is if the dose is lowered the effectiveness of the dilution may be lessened, leading to selection being greater and resistance increasing over time. However this was only found in a very small parameter space. In general reducing the dose to 50% of a full dose did not lead to loss of suppression and more often led to suppression being achieved as selection was reduced more than the dilution effect was decreased.

Additionally our model demonstrates that sexual reproduction is not required to maintain a low resistance allele frequency, and, without immigration, a sexual population cannot maintain a low resistant allele frequency no matter how high the dose. Therefore the reasoning presented in Georghiou and Taylor (1977) - that a high dose that can kill all heterozygote individuals will prevent the resistance from building up by ensuring the resistance is maintained in the heterozygote form - despite being seemingly intuitive, is fallacious; in the model presented here a substantial influx of untreated individuals is needed to ensure resistance does not increase, with no emigration back into the untreated individuals, and this is not dependent on the reproduction system being sexual.

When resistance does not remain low indefinitely, the question that needs addressing is whether lowering the dose of the insecticide will lead to resistance building up faster or slower. When there is no portion of the pest population that is untreated (whether an untreated population separate from the treated population, or a stage that is not susceptible to the insecticide), lowering the dose will always lead to a slower build-up of resistance. When a portion of the population is unaffected by the insecticide, it becomes possible for lowering the dose to increase the rate at which resistance builds up, and this is independent of the reproduction strategies. Again we attribute this to the trade-off between dilution and selection – when dose is reduced from a very potent full dose the dilution from susceptible individuals becomes significantly less, whereas there is still a high selection pressure even at 50% of the full dose.

These results suggest that using a high dose is very rarely an optimal resistance management strategy The main message from this paper is therefore that, where practically possible, insecticide dose should be reduced in order to preserve efficacy of the chemical. While this is a strong message based on several modelling studies (Georghiou and Taylor, 1977, Rosenheim and Tabashnik, 1990, Tabashnik and Croft, 1982), there is little experimental literature to back up these findings. No experimental papers have been found that attempt to test the dose hypothesis. It would be beneficial to have a larger experimental dataset to validate these modelling studies across a range of contrasting pest species and modes of action. The observational evidence available, to support or contradict the findings, comes from the wide range of resistance cases that have occurred in many pest species against many insecticide modes of action. Although it is a subjective judgement, it seems unlikely that all these cases have arisen because of poor compliance with the current guidance to adhere to the dose recommended on the label.

Although we have attempted to accurately simulate agricultural insect pest populations, values for some parameters are difficult to obtain. In particular, both the dominance of a resistance gene and the level of immigration into an insect population are difficult to determine, despite being critical parameters.

Our model has considered cases where a single target-site mutation confers a high level of resistance. Whilst this type of resistance has been the major focus of most insecticide resistance research and is a primary resistance mechanism in many pests, in some insect pests resistance is determined, at least in part, by several genes that each contribute toward the resistance, either through metabolic resistance (Espinosa et al., 2005, Riga et al., 2014) or by making the insect anatomically more resistant. Both the genetics and the resulting resistance phenotype may be very different from those analysed in this paper.

The work described here is analogous in some respects to work on resistance to transgenic crops. Theoretical modelling work (Sisterson et al., 2004) has suggested that a high-dose refuge strategy (utilising a dose high enough to kill all insects that are either susceptible or heterozygote, with a refuge containing purely susceptible individuals) can indefinitely suppress resistance. The results presented here are consistent with that theoretical outcome, assuming that a transgenic crop has a much higher efficacy than an insecticidal spray, and that the dilution from the refuge is high enough to dilute the selection for homozygote resistant individuals. However with field applications of insecticides the strategy proposed for transgenic crops seems unlikely to work, as the dose applied cannot be high enough to kill all susceptible individuals as well as heterozygotes.

One other factor has not been addressed in this paper, that of the cost of resistance, which is likely to affect the rate of resistance selection. We demonstrate in Appendix 4 that the inclusion of a fitness cost into the model results in selection developing slower as found in previous models (Rosenheim and Tabashnik, 1990), but their inclusion did not result in high doses being a better resistance management strategy than a reduced dose.

We are aware, and have tried to repeatedly emphasise, that the effect of dose on the effectiveness of control of a pest species has not been addressed in this paper. It is clear that an optimal resistance management strategy, considered in isolation from the practical need to obtain effective control, would be to apply zero dose, therefore applying no selection pressure to evolve. The strategies explored in this paper have only been compared with respect to their ability to prevent an increase in resistance, and not whether or not the insecticide application strategy controls the insect pest density adequately to protect the marketable yield of the crop. This extension would require a description of how insect density relates to yield and quality, and therefore what pest density would be tolerable. However the relationship between insect density and yield is a complex area, with many insects damaging the crop in different ways and at different times, whether through direct feeding damage of one or more stages of the insect, through product contamination or by introducing viruses. Each of these damage mechanisms requires a different density / damage model. The trade-off between selection and effectiveness of control should be explored explicitly by the approach described above.

## 5 Conclusions

Our results suggest that, in most plausible circumstances for target-site resistance, the effective life of insecticide modes of action would be increased by reducing insecticide dose below the maximum permitted, where that can be done without prejudicing effective control.

## Figures and Tables

**Table A1.1:** A punnett square. The two parents’ genotypes are in bold, and the proportion of each genotype from each parent cross is given in each internal square.

	SS	SR	RR
SS	1.0 SS	0.5 SS	0.0 SS
	0.0 SR	0.5 SR	1.0 RS
	0.0 RR	0.0 RR	0.0 RR
SR	0.5 SS	0.25 SS	0.0 SS
	0.5 SR	0.5 SR	0.5 SR
	0.0 RR	0.25 RR	0.5 RR
RR	0.0 SS	0.0 SS	0.0 SS
	1.0 SR	0.5 SR	0.0 SR
	0.0 RR	0.5 RR	1.0 RR

**Fig. D.1:**
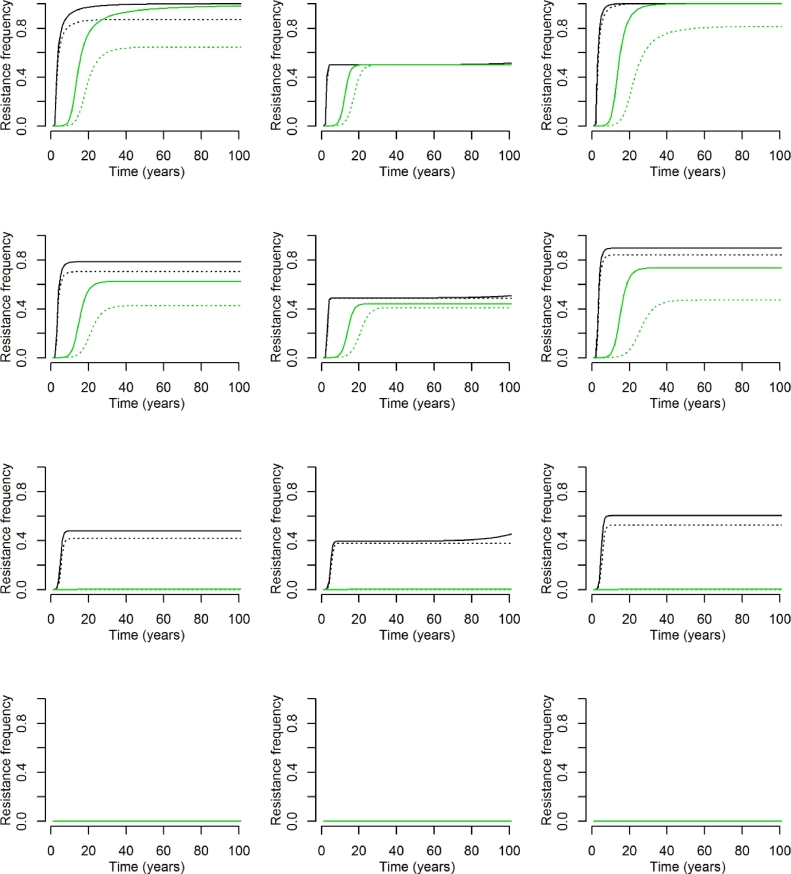
The resistance frequency of a population when challenged with a full dose of insecticide (black) and a half dose of insecticide (green), when the insect population in question has no fitness cost (solid line) or a 10% fitness cost (FC=0.1) to being resistant (dashed line). The left hand column represents a sexually reproducing diploid population; the middle column an asexually reproducing diploid population; and the right column a sexually reproducing haplodiploid population. The top row has no immigration or emergence; and each subsequent row contains simulations from an insect population with higher immigration (low ($\iota_{I}=0.001$), medium ($\iota_{I}=0.01$) and high ($\iota_{I}=0.1$)), but no emigration ($\iota_{E}=0.0$).

**Fig. D.2:**
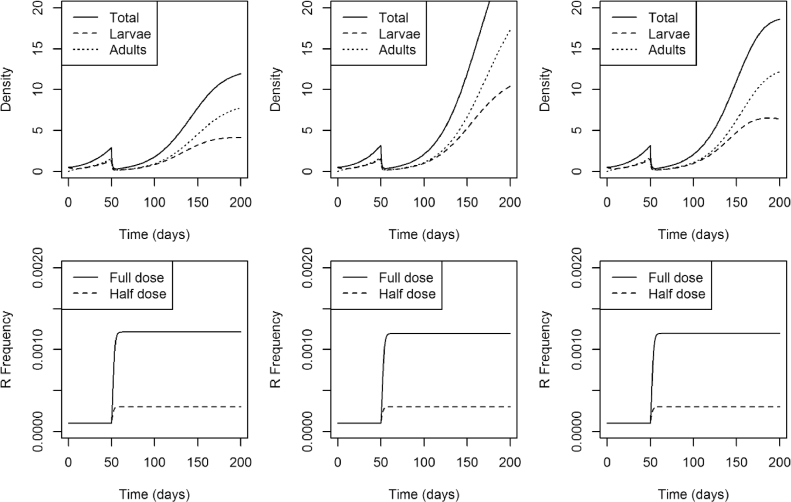
Changing the life stage of the insect that affects the density dependence does not affect the rate at which resistance is selected for. In the left column, the model is formulated so that the birth rate is dependent on the density of both adults and larvae in the population; in the central column the birth rate is dependent on the density of adults; in the right hand column the birth rate is dependent on the density of larvae. The carrying capacity in each case is the same. The top row shows the population density of adults and larvae when a single full dose of insecticide is applied, while the bottom row shows the resultant change in resistance in the population with both a full and half dose applied. Other parameters are based on the default model (see Table 1).

## Appendix A Parameterization of each insect species

### A.1 Myzus persicae

The peach-potato aphid is a multivoltine pest of many arable and greenhouse crops. The insect is viviparous, and so only larvae (nymphs) and adults are modelled. Life cycle characteristics are highly temperature-dependent, however a mean summer temperature of 15 degrees was assumed, resulting in a mean lifespan of 7 days and 10 days for larvae and adults respectively, and the adults having a birth rate of 1.5 larvae per day per female (Harrington and Xia-Nian, 1984), and natural mortalities of 0.2 for both adults and larvae. The insects overwinter as larvae, and are assumed to emerge from this population in an average of 10 days. The species is predominantly parthenogenetic in temperate regions (Blackman, 2009), and so is assumed to be asexual here. Mortality for larvae and adults are based on kdr (‘knock-down resistance’ ) to the pyrethroid deltamethrin. The gradient (*b*) of the logit-dose line is assumed to be the same for each genotype, 2.0, while the intercept (*a*) is 2.5 for the SS genotype and −0.5 for the RR genotype (Martinez-Torres et al., 1999), with allele dominance of 0.45. The season over which *M. persicae* is active was assumed to be 200 days, and a single spray was applied on day 50.

### A.2 Meligethes aeneus

The pollen beetle is an important pest of oilseed rape in Europe. It is a univoltine, sexually reproducing beetle. The beetle overwinters in its adult stage outside the crop, before migrating into the crop in early spring, where the adults feed on unopened buds before the flowers have opened, damaging yields. The adults lay eggs inside unopened buds where the larvae feed causing additional damage. Development rates are dependent on temperature, and therefore approximate values are used for a representative environment. The lifespan of each stage is: Egg = 5 days; Larvae = 20 days; Pupae = 10 days; Adults = 12 days. Females were assumed to lay 4 eggs per day (Ferguson et al., 2015), giving a birth rate of 2 eggs per day per beetle. Daily per-capita mortality rates were calculated from the percentage survival of each stage in (Nielsen and Axelsen, 1988) and converted to mortality rates, giving 0.01 and 0.025 per egg and larva respectively. Mortality of pupae was determined from (Büchi, 2002) giving a mortality rate of 0.01 per pupa. Data on the natural mortality of adults was not found, and so was set as 0.01. While kdr-resistance to pyrethroids is known in pollen beetles LC50s are not given, however 100% kdr-resistance does confer full resistance to *lambda*-cyhalothrin (Nauen et al., 2012). We therefore assumed a gradient of 2.0, and that resistance confers a 1000 fold resistance ratio. The season over which *M. aeneus* was modelled was 60 days, with a single spray on day 5, as on oilseed rape early sprays are important to protect an early crop from damage to unopened buds.

### A.3 Frankliniella occidentalis

Western flower thrips is a polyphagous multivoltine haplodiploid insect affecting many greenhouse crop species. The males are haploid and are produced from unfertilised eggs, and the females are diploid resulting from fertilised eggs. We parameterised the model based upon the insect feeding on cucumber (*Cucumis sativus*) at 20 °C, from (Gaum et al., 1994). The lifespan of each stage is therefore: 6.7, 9.8, 5.2 and 25.2 days on average for the eggs, larvae, pupae and adults respectively. The birth rate was assumed to be independent of density, as this insect can grow exponentially to very high levels, and was set to be 8.6 per adult female during her lifespan, of which 50% became male, and 50% became female. Only one target-site resistance mechanism is currently confirmed in *F. occidentalis*, that of resistance to spinosad (Bielza et al., 2007). However an LC50 for the resistant strain was not given, only that the resistance ratio was, in the most extreme case, > 13,500 (Bielza et al., 2007). Therefore we assumed a resistance factor of 10^5^ in our simulation and a logit-dose gradient of 1.0. The insecticide is similar efficacious for adults and larvae (Jones et al., 2005). Each season was modelled for 70 days, the maximum length possible before the computational limit of the density of the insect in the absence of insecticide application was reached. A single spray was applied on day 20.

## Appendix B Implementation of sexual reproduction

The density of new larvae in a sexual population can be determined from the birth rate (*β*) multiplied by the density of all adult genotypes (*A_T_*). Additionally however the genotype proportions of these new larvae must be established. Therefore we need to estimate the proportion of the new larvae that are of genotype *G; p_G_*. The expected proportion of each genotype can be calculated with the use of a punnett square, which illustrates the proportion of each genotype resulting from any genotype cross according to independent assortment (Table A1.1).

The punnett square gives the proportion of genotypes from any combination of parent genotypes and this must be weighted by the actual densities of the adult genotypes in the population. Therefore in order to calculate the total proportion of a single offspring genotype, each parental cross is considered in turn (SS crossed with SS; SS crossed with SR; SS crossed with RR; etc…), and as each cross is considered, the proportion of each cross that results in the specified offspring genotype is multiplied by the density of each of adult genotypes that make up the cross. This is then converted into a proportion by dividing by the squared total adult density.

That is, when estimating the proportion of offspring *SR* resulting from a cross between two adults *SS* and *SR* the density of offspring resulting from the cross would be 0.5**A_SS_***A_SR_*. Dividing by *A_T_***A_T_* would therefore be the proportion of offspring from that cross that are of genotype *SR*.

Therefore the total proportion of offspring with genotype *SS* from all parental crosses may be calculated as:

$$p_{SS}=\frac{\begin{array}{c} 1*A_{SS}*A_{SS}+0.5*A_{SR}*A_{SS}+0*A_{RR}*A_{SS}\;\\ \;+\;0.5*A_{SS}*A_{SR}+0.25*A_{SR}*A_{SR}+0*A_{RR}*A_{SR}\\ \;+\;0*A_{SS}*A_{RR}+0*A_{SR}*A_{RR}+0*A_{RR}*A_{RR} \end{array}}{A_{T}*A_{T}}$$

This simplifies to:

$$p_{SS}=\frac{A_{SS}A_{SS}+A_{SR}A_{SS}+0.25A_{SR}A_{SR}}{A_{T}A_{T}}$$

## Appendix C Derivation of the logit-dose model

Experimental insecticide resistance literature typically provides LC_50_ values and slopes for insecticides, as it was early on recognised that the probit of insect mortality is linearly related to the logarithm of the applied insecticide dose concentration. Instead of the probit function, we have used the logit function to transform mortality, a very similar function that also linearises the mortality log dose data, but which is easier to work with.

Given that the logit of mortality is a straight line we can write:

logit(m)=a+blog(D)

where *m* is mortality over a given time period (typically either 24 h or 48 h), and *D* is the dose applied.

This is equivalent to:

$$\text{log}\left(\frac{m}{1-m}\right)=a+b\;\text{log}\left(D\right)$$

Rearranging for *m*:

$$m=\frac{10^{a+b\log\left(D\right)}}{1+10^{a+b\log\left(D\right)}}=\frac{10^{a}D^{b}}{1+10^{a}D^{b}}$$

We assume that the decline in population following an application of insecticide can be specified by a single dose-dependent mortality rate. Therefore, if starting with an initial population density of *N*_0_, we expect the density of the insect population (*N*) at any time, *t*, to be:

$$N\left(t\right)=N_{0}e^{-\delta t}$$

where *δ* represents the rate of decrease of the population.

Re-writing the above equation as the proportion surviving 1 day: $\frac{N(t)}{N_{0}}=e^{-\delta t}$, and recognising that the proportion surviving is 1–the proportion dying, then:

$$m=\frac{10^{a}D^{b}}{1+10^{a}D^{b}}=1-\frac{N\left(t\right)}{N_{0}}=1-e^{-\delta t}$$

Rearranging to estimate the mortality rate, *δ*:

$$\delta =-\frac{1}{t}\ln\left(1-\frac{10^{a}D^{b}}{1+10^{a}D^{b}}\right)=\frac{1}{t}\ln\left(1+10^{a}D^{b}\right)$$

*t* is 1 day, and so may be removed from the equation.

Therefore, in the differential equations, the rate of change of any population of insects (*N*) as a result of an insecticide dose may be determined by:

$$\frac{dN}{dt}=-\ln\left(1+10^{a}D^{b}\right)N$$

and so is determined by the dose of insecticide applied (*D*), and the intercept (*a*) and slope (*b*) of the logit-dose curve.

## Appendix D Further parameter searches

### D.1 Fitness cost

We state in the discussion that the addition of a fitness cost of being resistant is unlikely to change the qualitative results presented in the paper, specifically that in the vast majority of insect-insecticide systems lowering the dose of insecticide applied will lead to a reduction in the selection rate. In this section we attempt to demonstrate this.

We assume that the fitness cost of resistance decreases the birth rate of the insect. Specifically, a 10% fitness cost will lead to a 10% reduction in the per capita birth rate. That is $\beta_{RR}=\left((1-FC)\cdot \beta \right)$, where *FC* is the percentage fitness cost. We additionally assume that the fitness cost of heterozygote individuals is determined by the dominance of the resistance gene, such that a fully recessive gene will lead to individuals of genotype SR having the same birth rate as the SS individuals, and so on.

Fig. D.1 demonstrates the result of lowering the dose of insecticide from a full dose (for an insecticide with 99% efficacy) to a half dose for each reproduction mode (from left to right, sexual, asexual, haplodiploid), and different levels of immigration (from top to bottom, none to high). The resistance frequency is shown for populations with and without a 10% fitness cost. Although the addition of a fitness cost does result in slower selection and resistance suppression at lower levels of immigration, in all cases where resistance developed applying a half dose of insecticide results in slower selection for resistance than when a full dose of insecticide is applied. Results were also tested with emigration, with the same qualitative result.

### D.2 Density dependence

Different insects may be constrained by different stages of their life cycle. Here we demonstrate that the dependence of the birth rate on the density of different life cycle stages does not affect the conclusions presented in the paper. Fig. D.2 demonstrates three different density dependence assumptions on the generic parameterisation of the model. In the first column the birth rate is adjusted as per the paper, wherein the birth rate of adults is constrained by both the density of the larvae and the adults in the pest population (being a hemimetabolous parameterisation, these are the only two stages). $\beta {\left(1-\frac{\sum_{GG}T_{GG}+\;\sum_{G}T_{G}}{K}\right)}^{+}$. In the second column the birth rate is dependent only on the density of the adults in the population, and in the third column the birth rate is dependent on the density of the larvae. In each case the population growth (top row) alters depending on which stage or stages are critical. However, on the second row of Fig. D.2 we demonstrate that this has little to no impact on the rate at which resistance is selected for. Upon application of a full or half dose of insecticide, the resistance builds up to the same degree in each case.
